# Leptin-Driven Distinct Transcriptional Reprogramming in Triple-Negative Breast Cancer: Implications for Racial Disparities

**DOI:** 10.3390/ijms27219680

**Published:** 2026-10-29

**Authors:** Sarabjeet Kour Sudan, Amod Sharma, Shashi Anand, Lavanya Challagundla, Jasleen Kaur, Barbara S. Craft, Ajay P. Singh, Seema Singh

**Affiliations:** 1 Department of Cell and Molecular Biology, University of Mississippi Medical Center, 2500 North State Street, Jackson, MS 39216, USA; 2 Cancer Center and Research Institute, University of Mississippi Medical Center, 2500 North State Street, Jackson, MS 39216, USA; 3 Division of Oncology, University of Mississippi Medical Center, Jackson, MS 39216, USA

**Keywords:** leptin, transcriptome, triple-negative breast cancer, obesity, metabolism, breast cancer disparities

## Abstract

Obesity is an established risk factor for breast cancer (BC), and leptin, an adipocyte-derived hormone, may link obesity to tumor progression. However, the role of leptin in the molecular heterogeneity of triple-negative breast cancer (TNBC), particularly across racial groups, remains unclear. Here, we examined circulating leptin levels in Black and White women with and without BC and characterized leptin-induced transcriptional changes in TNBC cell lines of White and Black racial backgrounds using RNA sequencing. Functional enrichment analyses were performed using Gene Ontology and KEGG pathway annotations, and clinical relevance was assessed using publicly available datasets. Circulating leptin levels were significantly elevated in women with BC and were relatively higher in Black than White women and associated with increased odds of luminal A and TNBC diagnoses. Leptin induced distinct transcriptional responses in two TNBC cell lines, with differential enrichment of oncogenic, metabolic, and signaling pathways, including spliceosome, steroid biosynthesis, PI3K-AKT, Notch, and extracellular matrix-receptor interaction. Several leptin-responsive genes showed concordant expression in clinical specimens and exhibited race- and subtype- associated differences and prognostic significance. Collectively, these findings identify distinct, context-dependent leptin-responsive transcriptional programs in TNBC and provide insight into their potential contributions to tumor heterogeneity and breast cancer disparities.

## 1. Introduction

Breast cancer (BC) is the most common malignancy in American women. In 2026, an estimated 321,910 new cases of invasive BC and 42,140 deaths are expected [1]. Triple-negative breast cancer (TNBC) is the most aggressive molecular subtype characterized by the absence of estrogen receptor (ER), progesterone receptor (PR), and human epidermal growth factor receptor 2 (HER2) expression. TNBC accounts for approximately 15–20% of all BC cases and is associated with poor prognosis, increased metastatic potential, early recurrence, and limited targeted therapeutic options [2]. Importantly, the burden of TNBC is disproportionately higher in Black women, who exhibit higher incidence rates, earlier disease onset, more aggressive clinical behavior, and poorer survival outcomes compared with White women [3,4]. Although socioeconomic and healthcare-related factors contribute to these disparities, growing evidence suggests that biological mechanisms also play a critical role in shaping TNBC progression and racial differences in disease outcomes [5,6].

Breast cancer is a molecularly heterogeneous disease characterized by alterations in gene expression that contribute to tumor progression, metastasis, and treatment response. Transcriptomic profiling has facilitated the identification of molecular signatures associated with tumor characteristics and clinical outcomes, complementing established prognostic and predictive biomarkers [7,8]. In addition to protein-coding messenger RNAs (mRNAs), dysregulation of non-coding RNAs, including microRNAs (miRNAs), long non-coding RNAs (lncRNAs), and circular RNAs (circRNAs), plays an important role in breast cancer biology by regulating gene expression and pathways involved in tumor growth, invasion, metastasis, and therapeutic resistance. Aberrantly expressed miRNAs, including miR-21, have been associated with breast cancer prognosis, while circulating miRNA panels show potential as diagnostic and prognostic biomarkers [9,10,11]. Similarly, ncRNA signatures, including subtype-associated lncRNAs in triple-negative breast cancer (TNBC), have emerged as potential prognostic biomarkers and therapeutic targets [12,13].

Obesity has emerged as a major risk factor for BC diagnosis and mortality, particularly in patients with aggressive BC subtypes [6,14,15]. We recently showed that it is also associated with an early onset of BC and a higher risk of diagnosis with luminal A or TNBC subtypes, especially in Black women [16]. Among obesity-associated factors, leptin, a multifunctional adipokine primarily secreted by adipose tissue, has gained considerable attention for its role in tumorigenesis. We observed higher serum leptin levels in Black women than in White women, with a positive association with patient body mass index (BMI) [16]. In addition to regulating appetite and energy homeostasis, leptin promotes cancer cell proliferation, angiogenesis, migration, stemness, inflammation, and resistance to apoptosis by activating several oncogenic signaling pathways, including JAK/STAT3, PI3K/AKT, and MAPK [17,18,19,20,21]. Therefore, leptin-mediated signaling may play a significant role in BC pathogenesis and contribute to racially disparate clinical outcomes.

In the present study, we expanded our prior observations by examining serum leptin levels in a larger cohort of patient samples and investigated the associations with disease-onset age and diagnosis with specific molecular subtypes. We also investigated the impact of leptin on the transcriptomic landscape in TNBC cell lines derived from Black and White patients to identify cell line-specific molecular responses and their impact on clinically relevant signaling pathways. Transcriptomic findings were integrated with clinical datasets to establish the translational significance of leptin-responsive changes in the cellular transcriptome. In addition, we examined racial and subtype-specific expression patterns and assessed the prognostic relevance of leptin-induced genes in TNBC patient cohorts. Collectively, this study provides important mechanistic insights into the role of leptin-mediated signaling in TNBC and highlights potential molecular targets that may contribute to BC disparities and disease progression.

## 2. Results

### 2.1. Leptin Levels Are Disproportionately Higher in Breast Cancer Patients

**Figure 1:**
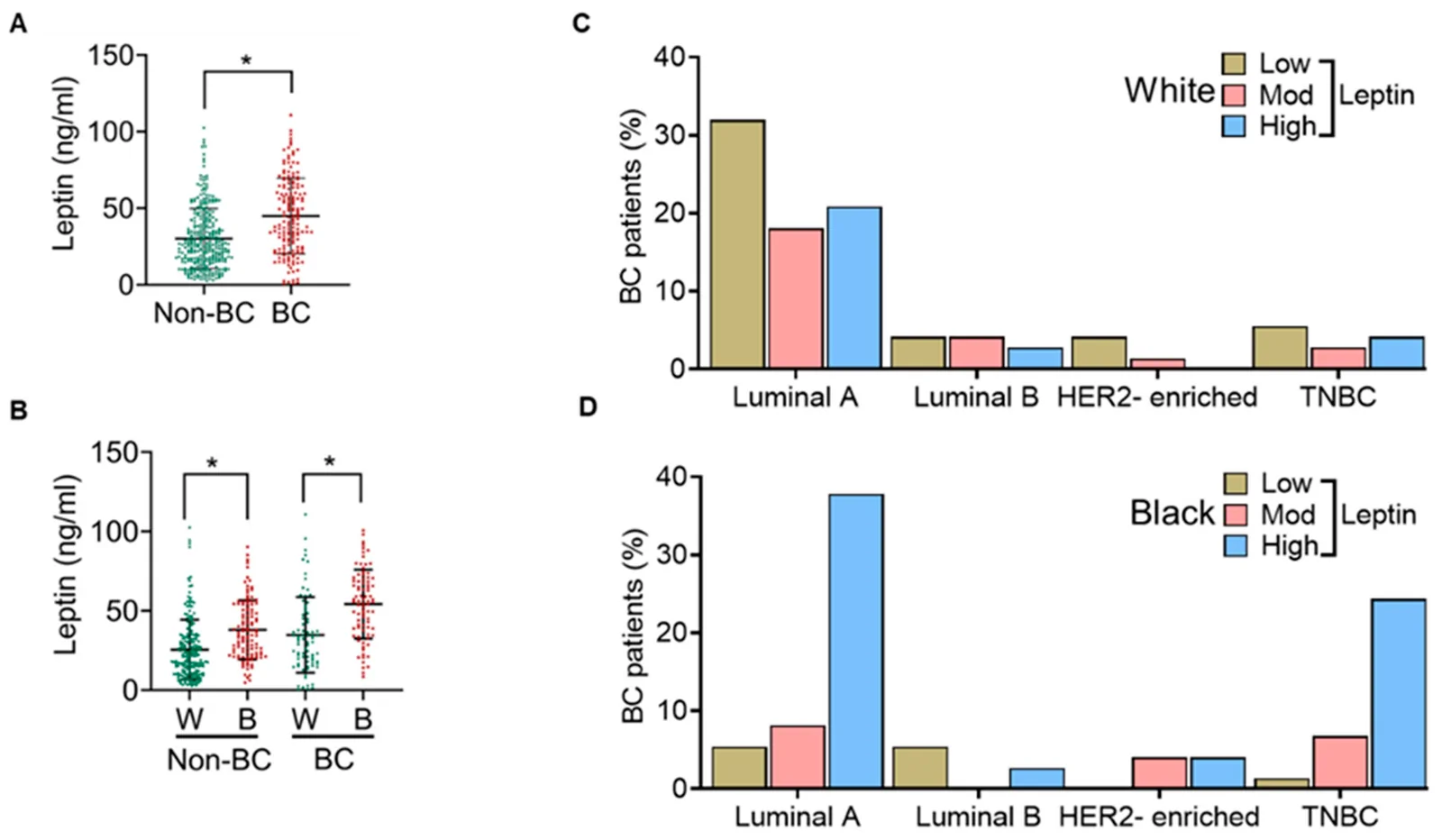
Serum leptin levels in BC and non-BC participants. (**A**) Serum levels of leptin in patients without BC (*N* = 306) and patients with BC (*N* = 162). Each dot represents an individual patient (**B**) Serum levels of leptin in Black and White women with BC (*N* = 84 Black and *N* = 78 White) and non-BC (*N* = 111 Black and *N* = 195 White). (**C**,**D**) Percentage distribution of BC subtypes among 3 leptin groups between White and Black women. TNBC indicates triple-negative BC. A *p*-value  <  0.05 was considered statistically significant and is indicated by asterisks (* *p* < 0.05).

Having observed an association of circulating leptin levels with BC risk and diagnosis across specific molecular subtypes [16], we expanded the study by recruiting additional subjects of different racial backgrounds: BC (84 Black and 78 White) and non-BC (111 Black and 195 White), and made consistent observations. We found significantly elevated serum leptin levels in women with a BC diagnosis (median [IQR], 42.2 [24.4–63.4] ng/mL) compared with the non-BC group (median [IQR], 26.2 [15.5–41.6] ng/mL) (*p*  <  0.001) (Figure 1A). Notably, Black women with or without BC exhibited significantly higher leptin levels (median [IQR], 56.0 [38.9–69.7] ng/mL and 34.8 [22.5–51.5] ng/mL, respectively) than White women (median [IQR], 31.7 [17.9–47.7] ng/mL and 21.5 [11.0–33.3] ng/mL, respectively) (*p* <  0.001) (Figure 1B). Next, we divided participants, based on the median leptin levels, into three categories [low, having leptin levels less than the median in women without BC (<26.2 ng/mL); high, leptin levels higher than the median in women with BC (>42.2 ng/mL); and moderate, when the levels were between these two medians (26.2–42.2 ng/mL)]. The data show that higher leptin levels are significantly associated with increased odds of being diagnosed with luminal A (OR, 6.7; 95% CI, 2.598–16.10; *p* < 0.001) and TNBC (OR, 6.00; 95% CI, 1.147–25.69; *p* = 0.04) in Black women (Figure 1C,D).

### 2.2. Leptin Induces a Distinct Set of Genes in Triple-Negative Breast Cancer Cells

**Figure 2:**
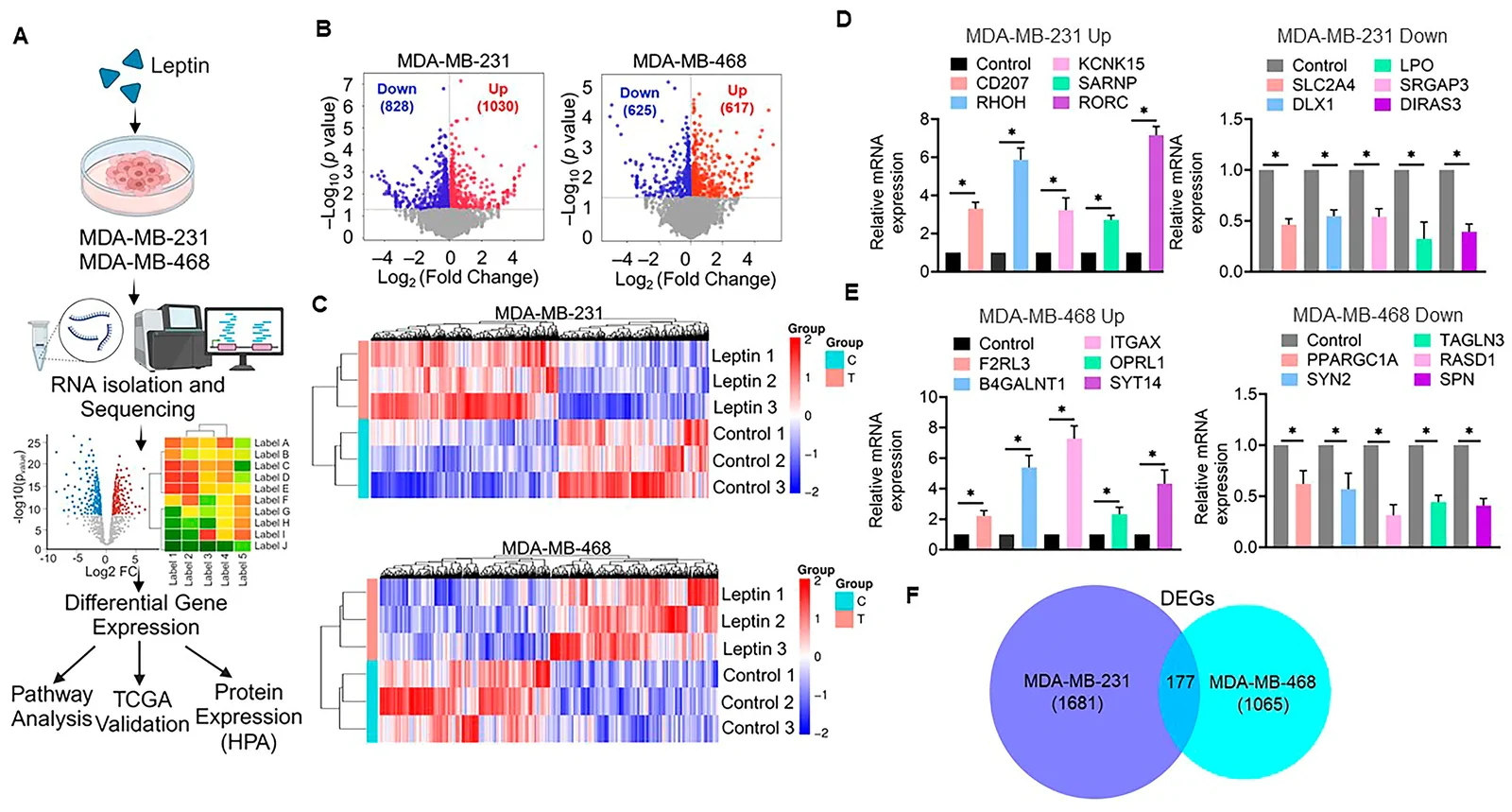
Leptin-induced transcriptional reprogramming in triple-negative breast cancer cells. (**A**) Schematic representation of the workflow. It was created using BioRender.com. (**B**) Volcano plots showing number of DEGs following leptin treatment in MDA-MB-231 and MDA-MB-468 cells. (**C**) Heatmaps of DEGs between vehicle-treated control and leptin-treated group in MDA-MB-231 and MDA-MB-468; rows represent samples and columns represent genes. (**D**,**E**) Validation of upregulated and downregulated genes in MDA-MB-231 and MDA-MB-468 cells. Bar graphs showing the relative expression levels of genes compared to vehicle-treated control. (**F**) Venn diagram showing the overlap of leptin-regulated DEGs between MDA-MB-231 and MDA-MB-468 cells. A *p*-value  <  0.05 was considered statistically significant and is indicated by asterisks (* *p* < 0.05).

To examine the impact of leptin signaling and whether that varies in a cell line- and racial origin-specific manner, we examined and compared the transcriptional landscape in vehicle (Tris-HCl) and leptin (100 ng/mL)-treated TNBC cell lines derived from Black (MDA-MB-468) and White (MDA-MB-231) (Figure 2A). Both cell lines express leptin receptor at comparable levels (Figure S1A) and demonstrate responsiveness to leptin treatment as examined by induction of STAT3 phosphorylation in a dose-dependent manner (0–200 ng/mL) (Figure S1B,C). The RNA-seq quality control metrics are provided in Table S1. RNA-seq data revealed robust transcriptional remodeling in both cell lines, with a substantial number of genes exhibiting significantly altered expression (*p* < 0.05). A total of 1030 and 617 genes were upregulated, and 828 and 625 were downregulated following leptin treatment in MDA-MB-231 and MDA-MB-468 cell lines, respectively (Figure 2B,C). These differential cell line-specific changes in gene expression were further validated by qRT-PCR in a randomly selected set of genes in MDA-MB-231 and MDA-MB-453 (derived from White patients), and MDA-MB-468 and HCC1806 (derived from Black patients) (Figure 2D,E and Figure S1D,E). Interestingly, in transcriptomics data, a large majority of genes were distinctly altered in MDA-MB-231 and MDA-MB-468 cells (Figure 2F). Collectively, these data show that leptin elicits heterogeneous transcriptional responses in TNBC cells, suggesting variable, cell-line-specific functional impacts on pathobiology.

### 2.3. Functional Enrichment Analysis Reveals a Diverse Leptin-Driven Pathway Remodeling in TNBC

**Figure 3:**
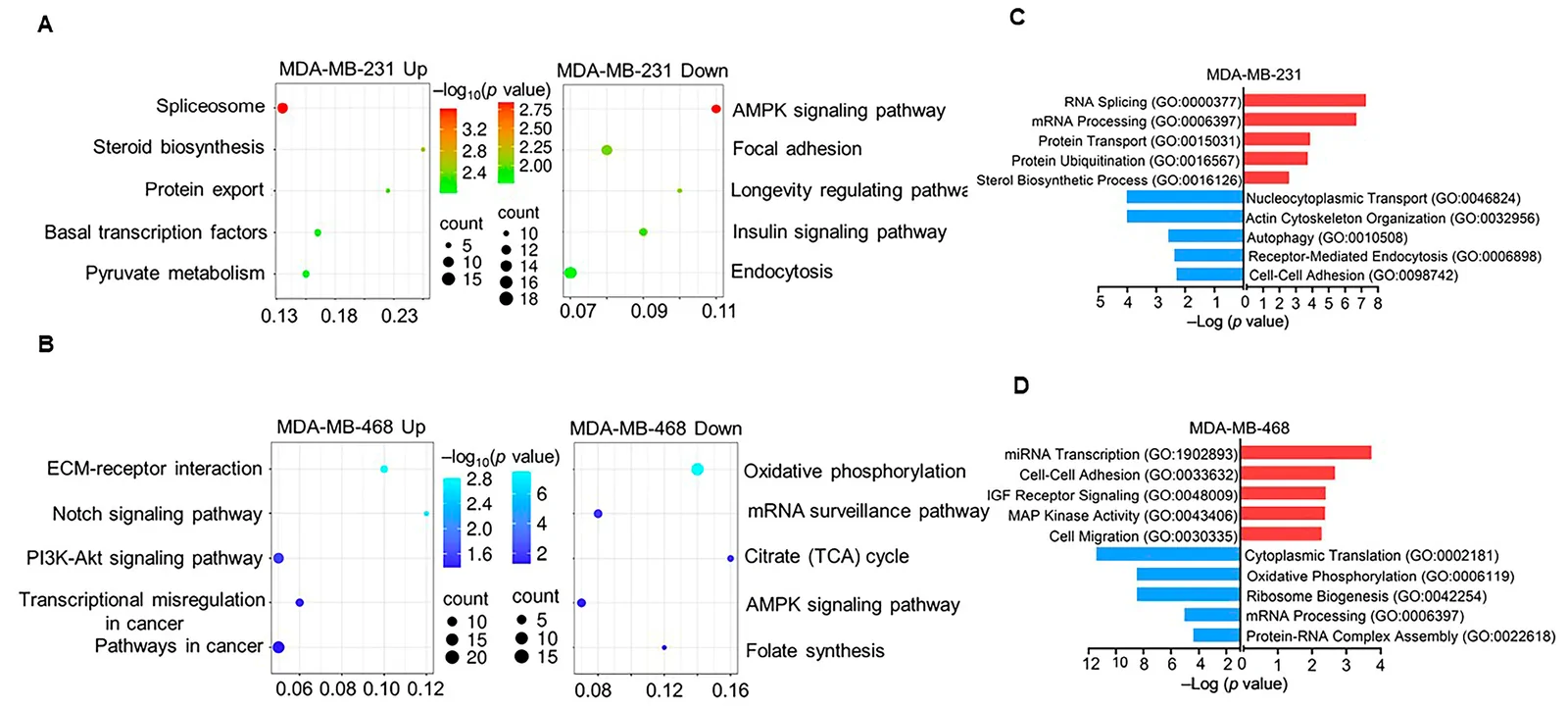
Pathway enrichment analysis of leptin-regulated genes in triple-negative breast cancer cells. (**A**,**B**) Bubble plots showing KEGG pathway enrichment analysis of DEGs, showing upregulated and downregulated pathways in MDA-MB-231 and MDA-MB-468 cells. Each bubble represents a pathway; bubble size reflects the number of differentially expressed genes contributing to that pathway, and color intensity indicates statistical significance. (**C**,**D**) Gene ontology analysis showing biological processes affected after leptin treatment in MDA-MB-231 and MDA-MB-468 cells compared to vehicle-treated control.

To elucidate the biological processes and signaling pathways regulated by leptin in TNBC, Gene Ontology (GO) and KEGG pathway enrichment analyses were performed using Enrichr on significantly upregulated and downregulated DEGs (*p* < 0.05) identified after leptin treatment. In MDA-MB-231 cells, KEGG enrichment analysis of significantly upregulated DEGs revealed a strong overrepresentation of pathways related to the spliceosome, steroid biosynthesis, protein export, basal transcriptional factors, and pyruvate metabolism. In contrast, significantly downregulated DEGs were enriched for the AMPK signaling pathway, focal adhesion, longevity-regulating pathway, insulin signaling pathway, and endocytosis (Figure 3A). In MDA-MB-468, KEGG enrichment of upregulated DEGs revealed pathways associated with ECM receptor interaction, Notch signaling pathway, PI3K-AKT signaling, transcriptional misregulation in cancer, and pathways in cancer, along with downregulation of oxidative phosphorylation, mRNA surveillance pathway, citrate cycle, AMPK signaling, and folate synthesis (Figure 3B). Genes associated with these pathways are provided in Table S2. Furthermore, GO analysis supported these findings, with upregulation and downregulation of processes associated with KEGG enrichment pathways in both cell types. In MDA-MB-231, GO enrichment of upregulated DEGs revealed processes involved in RNA splicing (GO:0000377), mRNA processing (GO:0006397), protein transport (GO:0015031), protein ubiquitination (GO:0016567), and steroid biosynthesis (GO:0016126), along with the downregulation of processes involved in nucleocytoplasmic transport (GO:0046824), actin cytoskeleton organization (GO:0032956), autophagy (GO:0010508), receptor mediated endocytosis (GO:0006898), and cell -cell adhesion (GO:0098742) (Figure 3C). In MDA-MB-468, GO processes shown significant upregulation of process related to mRNA transcription (GO:1902893), cell–cell adhesion (GO:0033632), IGF receptor signaling (GO:0048009), MAP kinase activity (GO:0043406), and cell migration (GO:0030335) along with the downregulation of processes involved in cytoplasmic translation (GO:0002181), oxidative phosphorylation (GO:0006119), ribosome biogenesis (GO:0042254), mRNA processing (GO:0006397), and protein-RNA complex assembly (GO:0022618) (Figure 3D). Additionally, protein–protein interactions among upregulated and downregulated pathway DEGs in both cell lines were analyzed using STRING v12.0 (Figure S2). In MDA-MB-231 and MDA-MB-468 cells, STRING analysis of upregulated DEGs revealed the presence of three and four dominant interaction clusters, respectively, whereas downregulated DEGs formed three and one major cluster in MDA-MB-231 and MDA-MB-468 cells, respectively. (Figure S2A–D). Genes associated with GO pathways are provided in Table S3. Together, GO and KEGG analyses demonstrate that leptin induces broad transcriptional reprogramming in TNBC cells, activating oncogenic and metabolic pathways. These data provide functional insight into leptin-driven signaling networks that may contribute to TNBC aggressiveness.

### 2.4. Clinical Validation of Leptin-Responsive Genes in TNBC

**Figure 4:**
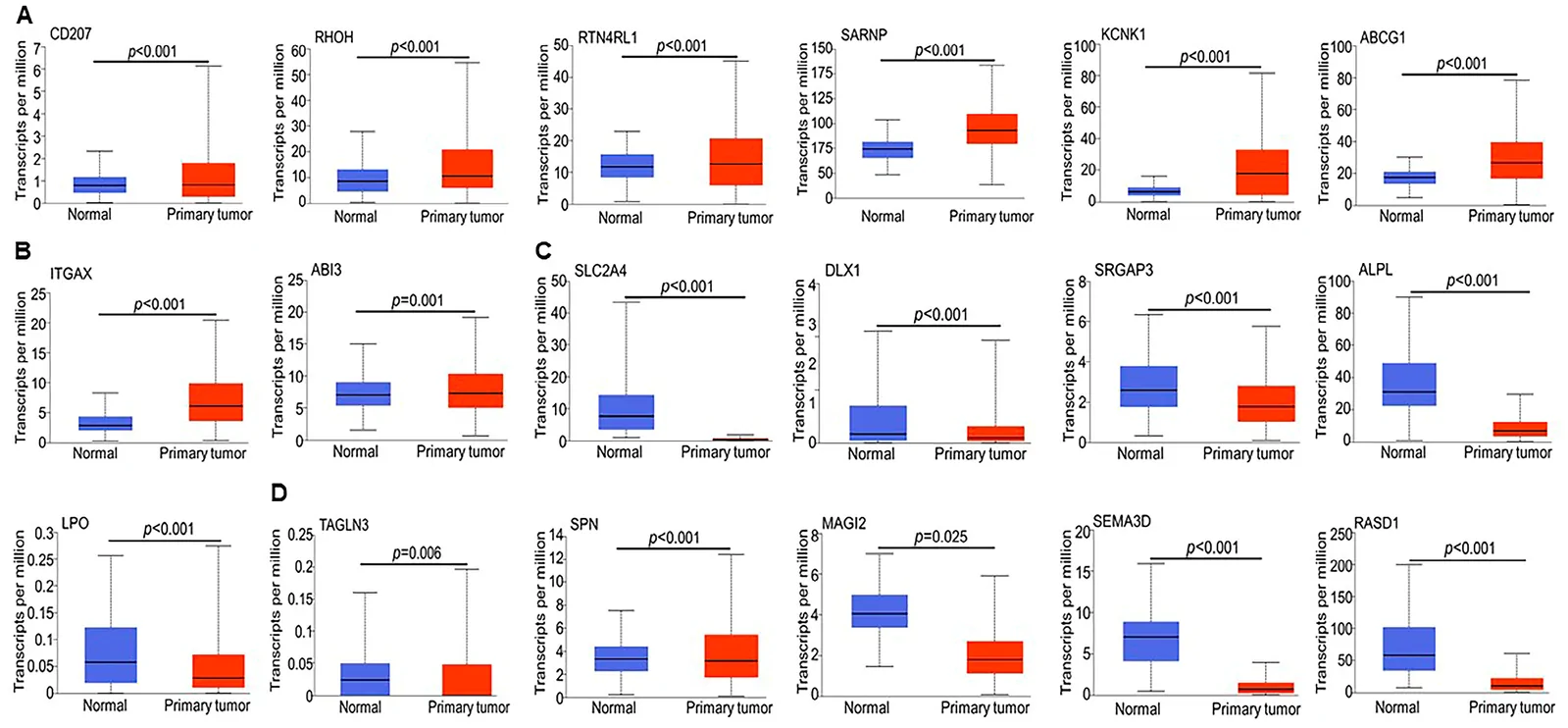
Differential expression of leptin-induced DEGs in TCGA-BRCA patient samples. (**A**,**B**) Box-and-whisker plots showing the expression levels of differentially expressed upregulated genes in MDA-MB-231 and MDA-MB-468 cells in tumor tissues (*N* = 1097) compared to normal counterparts (*N* = 114) using TCGA-BRCA dataset. (**C**,**D**) Box-and-whisker plots showing the expression levels of differentially expressed Downregulated genes in MDA-MB-231 and MDA-MB-468 cells in tumor tissues (*N* = 1097) compared to normal counterparts (*N* = 114) using TCGA-BRCA dataset. Statistical significance for tumor versus normal comparisons is indicated above each plot, with exact *p*-values.

We next analyzed TCGA BC RNA-sequencing data from the TCGA-BRCA cohort using UALCAN to evaluate the clinical associations of the top 50 significant up- and downregulated leptin-induced DEGs. This integrative approach enabled comparison of leptin-responsive genes observed in vitro with gene expression profiles derived from patient tumor samples. Notably, several genes that were significantly upregulated upon leptin exposure in cancer cells were also overexpressed in tumor tissues (*N* = 1097) compared with normal counterparts (*N* = 114). Numerous genes (*CD207*, *RHOH*, *RTN4RL1*, *SARNP*, *KCNK1*, *ABCG1*, *KCNK15*, *RORC*, *DNAI1*, *LHX2*, and *TMEM105*) induced by leptin in MDA-MB-231, were also significantly upregulated in tumor tissues (Figure 4A and Figure S3A). Further, leptin-induced genes in MDA-MB-468 (*ITGAX*, *ABI3*, *F2RL3*, *B4GALNT1*, *OPRL1*, *SYT14*, and *NINJ2*) were also significantly upregulated in tumor tissues (Figure 4B and Figure S3B). Similarly, genes downregulated following leptin treatment showed reduced expression in clinical cases such as *SLC2A4*, *DLX1*, *SRGAP3*, *ALPL*, *LPO*, *GNA14*, *COL23A1*, *TMPRSS3*, *DIRAS3*, *DCHS2*, *LRRC37A2*, *HAAO*, *POM121L9P*, and *ATP8B4* in MDA-MB-231 (Figure 4C and Figure S4A) and *PPARGC1A*, *TAGLN3*, *SPN*, *SH2D1B*, *VEGFC*, *MAGI2*, *SEMA3D*, *SYN2*, *RASD1*, *SLC16A12*, *PTH1R*, *TRIM68*, *HSPB8*, and *CYP11A1* in MDA-MB-468 (Figure 4D and Figure S4B). Together, these observations suggest that leptin alters the expression of several pathobiologically relevant genes that are aberrantly expressed in BC.

**Figure 5:**
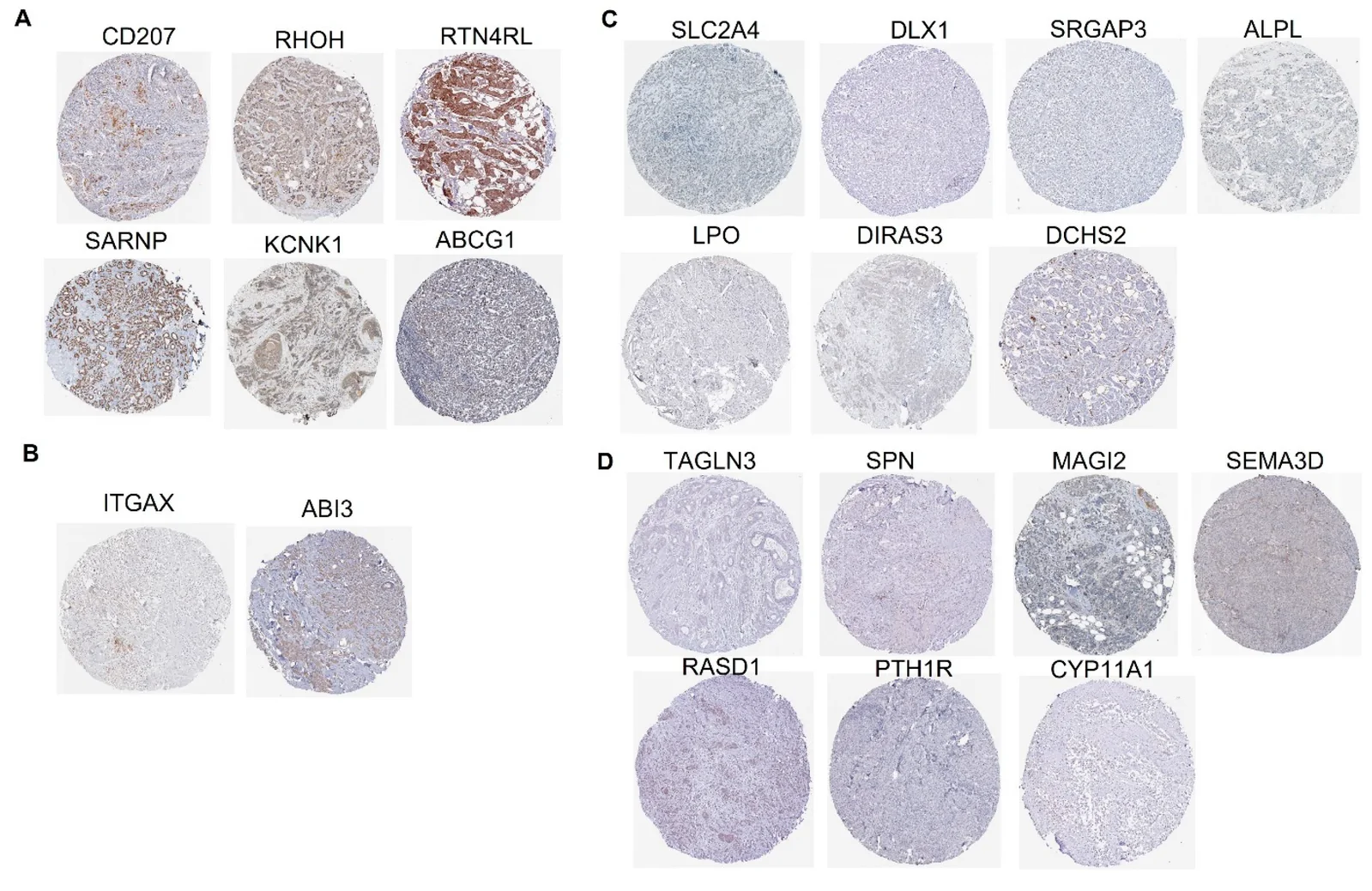
Immunohistochemical validation of selected leptin-responsive genes in breast cancer tissues. (**A**,**B**) Representative immunohistochemistry (IHC) images showing high protein expression patterns of selected leptin-responsive upregulated genes in breast cancer tissues using publicly available datasets (Human Protein Atlas). (**C**,**D**) Low protein expression patterns of selected leptin-responsive downregulated genes.

To assess whether leptin-induced transcriptional changes are reflected at the protein level, protein expression profiles of DEGs were examined using the Human Protein Atlas (HPA). Representative proteins corresponding to DEGs that were significantly upregulated following leptin treatment in MDA-MB-231 and MDA-MB-468 cells are shown in Figure 5A,B, as evidenced by increased staining intensity in BC tissues. Also, proteins encoded by downregulated DEGs in both cell types exhibited reduced expression levels, demonstrating concordance between transcriptomic and proteomic alterations following leptin treatment (Figure 5C,D). These consistent expression patterns across experimental and patient-derived datasets strengthen the biological and clinical significance of the identified leptin-responsive genes. Overall, the integration of transcriptomic data with Human Protein Atlas protein expression profiles demonstrates that leptin-induced changes in gene expression in TNBC cells are accompanied by corresponding alterations at the protein level, highlighting the biological relevance of leptin-responsive DEGs.

### 2.5. Race- and Subtype-Specific Association of Up- and Downregulated Differentially Expressed Genes

**Figure 6:**
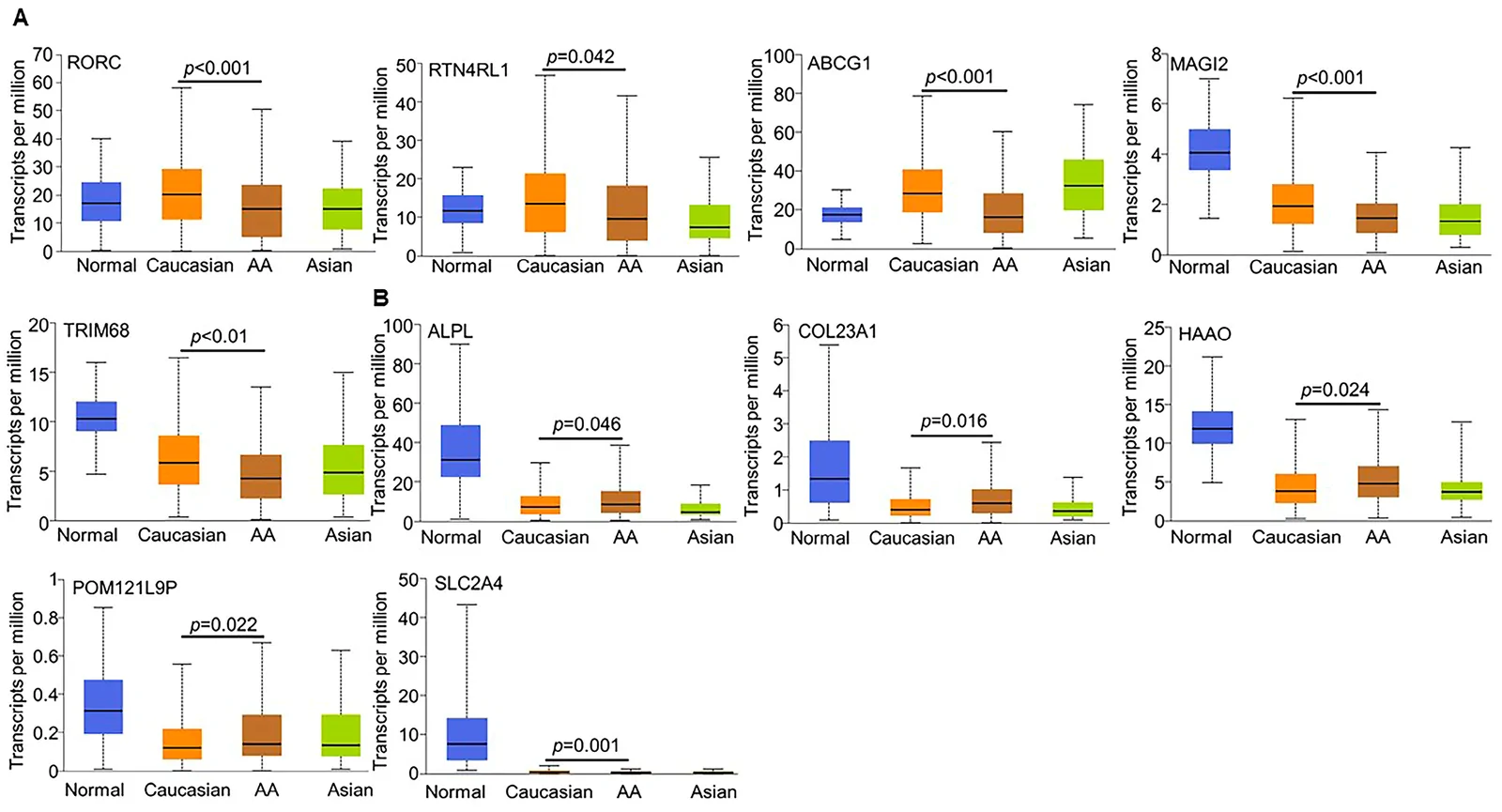
Race-specific expression of selected leptin-responsive genes. Box-and-whisker plots showing the RNA expression levels of leptin-responsive genes stratified by race using the TCGA-BRCA dataset. Expression profiles are shown for normal breast tissues (*N* = 114) and breast cancer samples from Caucasian (*N* = 748), African American (AA; *N* = 179), and Asian (*N* = 61) patients. (**A**) Expression of leptin-induced genes that are upregulated in Caucasian compared to AA breast cancer samples. (**B**) Expression of leptin-induced genes that are downregulated in Caucasian compared with AA breast cancer samples. Statistical significance for tumor versus normal comparisons is indicated above each plot, with exact *p*-values.

To investigate race-associated transcriptional differences, leptin-responsive DEGs identified in MDA-MB-231 and MDA-MB-468 cells were examined in the TCGA-BRCA cohort, including normal breast tissues (*N* = 114) and tumors from Caucasian (*N* = 748), African American (AA; *N* = 179), and Asian (*N* = 61) patients. Several leptin-regulated genes displayed significant race-specific expression patterns across patient groups (Figure 6). Among genes upregulated following leptin treatment in MDA-MB-231 cells, *RORC*, *RTN4RL1*, and *ABCG1* exhibited significantly lower expression in AA tumors compared with Caucasian tumors (Figure 6A). Similarly, *MAGI2* and *TRIM68*, which were downregulated in leptin-treated MDA-MB-468 cells, were expressed at significantly lower levels in AA patients than in Caucasians. In contrast, several genes demonstrated higher expression in AA tumors relative to Caucasian tumors (Figure 6B). These included *ALPL*, *COL23A1*, *HAAO*, *POM121L9P*, and *SLC2A4*, leptin-downregulated genes in MDA-MB-231 cells. Collectively, these findings demonstrate that a subset of leptin-responsive genes identified in TNBC cell lines exhibits race-associated expression patterns in BC patient tissues, suggesting that leptin-regulated transcriptional programs may differentially contribute to tumor biology across racial groups.

To assess the clinical relevance of the cell-derived DEGs, their expression patterns were subsequently compared with TNBC tissue samples using the UALCAN platform, which integrates TCGA-BRCA transcriptomic data. We observed that the top 50 significant DEGs with high fold change values identified in the TNBC cells (MDA-MB-231 and MDA-MB-468) after leptin treatment exhibited consistent expression trends in TNBC tissues (*N* = 116) compared to normal breast tissue (*N* = 114). Specifically, genes identified as upregulated in MDA-MB-231 and MDA-MB-468 cells showed elevated expression in TNBC patient tissue samples, whereas downregulated genes showed concordant reductions in expression, indicating strong agreement between in vitro and tissue-level data. Among the upregulated DEGs derived from MDA-MB-231 and MDA-MB-468 cells, genes that were consistently and significantly upregulated in TNBC tissues included *CD207*, *SARNP*, *LHX2*, *KCNK1*, and *TMEM105* (MDA-MB-231), and *F2RL3*, *B4GALNT1*, *ITGAX*, *NINJ2*, and *ABI3* (MDA-MB-468) (Figure S5A,B). Conversely, *SLC2A4*, *SRGAP3*, *GNA14*, *DIRAS3*, *DCHS2*, *LRRC37A2*, and *ATP8B4* (MDA-MB-231) and *VEGFC*, *MAGI2*, *SEMA3D*, *SYN2*, *RASD1*, *SLC16A*, *PTH1R*, TRIM68, *HSPB8*, and *CYP11A1* were significantly downregulated in TNBC tissues compared to normal (Figure S6A,B). Collectively, these findings demonstrate a high degree of concordance between TNBC cell-derived DEGs and patient-derived TNBC tissue expression profiles, supporting the biological relevance of these genes in TNBC pathogenesis.

### 2.6. Leptin-Induced Genes Have Prognostic Significance in Breast Cancer

**Figure 7:**
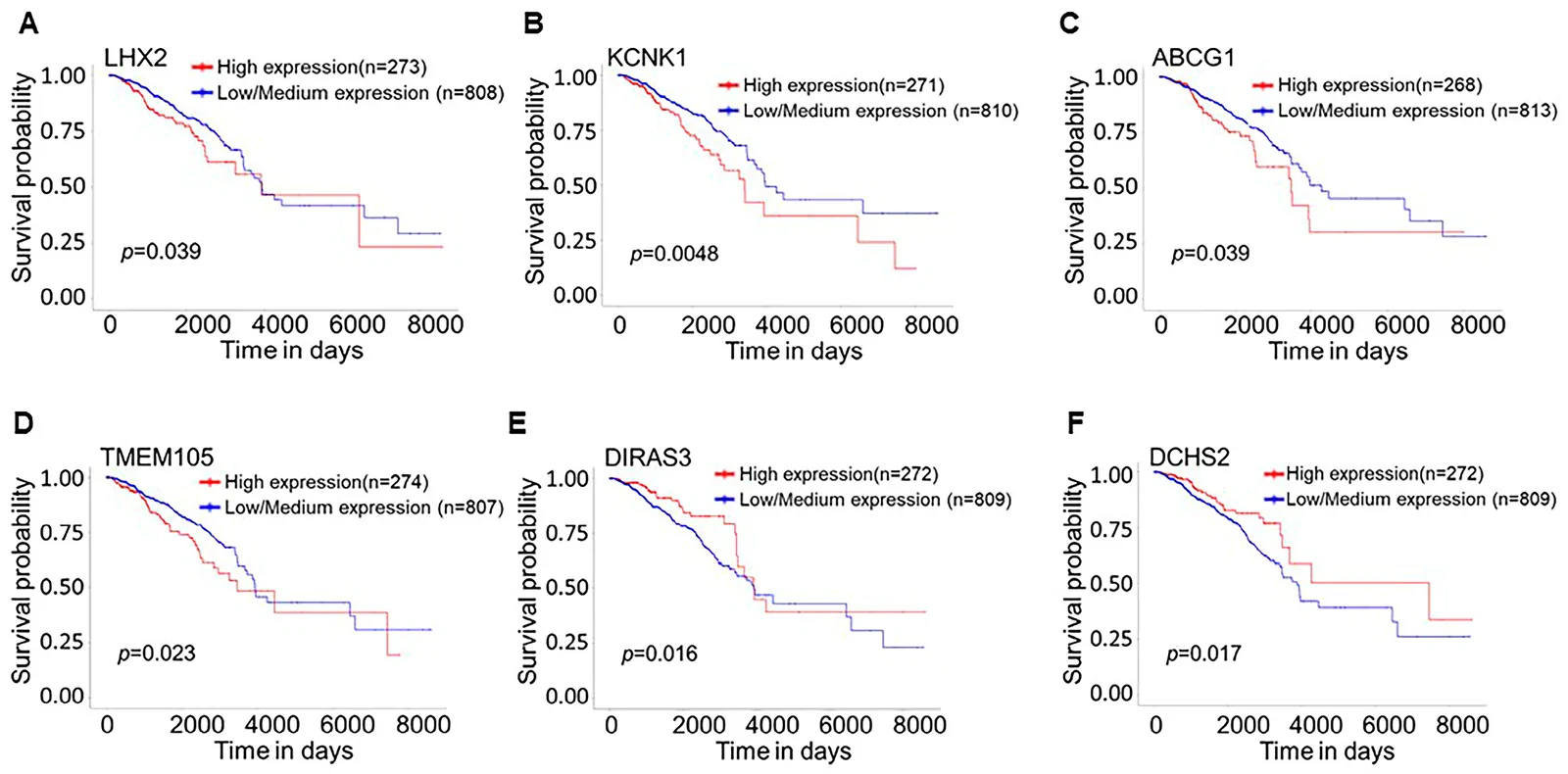
Survival outcomes with leptin-responsive differentially expressed genes in breast cancer patients. Kaplan–Meier survival analyses showing overall survival of breast cancer patients stratified by high versus low/medium expression of selected leptin-responsive genes using TCGA-BRCA cohort data: (**A**) *LHX2*, (**B**) *KCNK1*, (**C**) *ABCG1,* (**D**) *TMEM105,* (**E**) *DIRAS3,* (**F**) *DCHS2*. Statistical significance is indicated for each plot, with exact *p*-values.

To evaluate the prognostic relevance of leptin-responsive genes, we examined the correlation between overall survival and DEGs using the TCGA-BRCA cohort. Survival analysis revealed that several leptin-responsive genes were significantly associated with outcomes in BC patients. Genes upregulated following leptin treatment in MDA-MB-231, including *LHX2*, *KCNK1*, *ABCG1*, and *TMEM105*, showed a significant association with poorer overall survival of BC patients (*p* < 0.05), suggesting their potential roles in disease progression and aggressive tumor behavior (Figure 7A–D). In contrast, higher expression of selected genes that were downregulated upon leptin treatment, including *DIRAS3* and *DCHS2* (MDA-MB-231, down), correlated with improved patient survival, indicating possible tumor-suppressive functions (Figure 7E,F). Collectively, Kaplan–Meier survival curves demonstrated clear separation between high- and low-expression groups for multiple DEGs, with statistically significant differences in survival probability. The high-expression group includes samples with gene expression values equal to or higher than the 3rd quartile value, and a Low/Medium expression group includes samples with gene expression values less than the 3rd quartile [22]. These findings indicate that altered expression of leptin-responsive genes has measurable clinical implications in TNBC. Collectively, the survival analysis underscores the prognostic significance of DEGs identified following leptin treatment in TNBC cells. The concordance between in vitro expression changes and patient survival outcomes supports the potential utility of these genes as prognostic biomarkers and highlights the contribution of leptin-associated signaling pathways to TNBC disease progression.

## 3. Discussion

Among obesity-associated factors, leptin has emerged as a critical endocrine and paracrine mediator that promotes tumor growth, invasion, metastasis, angiogenesis, stemness, and therapeutic resistance [23,24,25,26,27]. Although numerous studies have demonstrated the oncogenic effects of leptin in BC, a comprehensive understanding of the global transcriptional programs regulated by leptin and their clinical significance remains limited. In this study, we examined circulating leptin levels in cancer and non-cancer subjects of different racial backgrounds and their association with specific subtype diagnoses. We also combined leptin-induced transcriptomic profiling with multiple independent patient datasets to define leptin-responsive gene networks in TNBC and establish their biological and clinical relevance. Our findings demonstrate that leptin induces extensive yet highly heterogeneous transcriptional remodeling in TNBC cells, activates multiple oncogenic signaling pathways, and regulates genes whose altered expression is recapitulated in breast tumors, exhibits race-associated differences, and predicts patient outcome. Collectively, these observations provide new insight into the molecular mechanisms through which obesity-associated leptin signaling may contribute to TNBC progression.

Consistent with our previous epidemiological observations [16], circulating leptin levels remained significantly associated with BC risk after expanding the patient cohort, particularly among Black women. Moreover, we observed an association between higher leptin levels and luminal A and TNBC diagnoses in Black women. These findings further support leptin as a clinically relevant obesity-associated biomarker and reinforce previous reports linking elevated leptin to increased BC risk and worse outcomes [24,28,29]. However, it should be noted that these findings do not account for potential confounding factors, such as age, metabolic disease, and adiposity, that may also influence circulating leptin levels. Future studies with larger cohorts and comprehensive adjustment for relevant clinical and metabolic factors are, therefore, warranted to further strengthen these relationships. Importantly, our findings also emphasize that obesity-associated endocrine alterations may disproportionately influence BC development in populations already experiencing a higher burden of aggressive disease. Although the present study was not designed to establish causality between elevated leptin and racial disparities, the observed associations highlight the importance of understanding leptin-mediated molecular pathways within the context of cancer disparities.

A major finding of this study is the remarkable heterogeneity of leptin-induced transcriptional responses between MDA-MB-231 and MDA-MB-468 cells. Despite both representing TNBC, only a relatively small proportion of differentially expressed genes overlapped between the two models. These observations revealed the molecular diversity of TNBC and suggest that leptin signaling is highly context-dependent, influenced by the underlying genomic and epigenomic landscape of individual tumors. Such heterogeneity likely reflects differences in downstream signaling circuitry, mutational profiles, and lineage-specific transcription factor networks [30,31,32,33,34]. Importantly, these findings caution against viewing leptin as regulating a single conserved transcriptional program and instead suggest that obesity-associated signaling converges on distinct molecular pathways depending on tumor context. A comparison of leptin-induced genes with those induced by other stressors, such as oxidative stress, using a publicly available database [Atlas of Stress Response Activity (ASTRA)] shows some overlap, suggesting that some of these changes may be shared with other cellular stress responses [35].

Functional enrichment analyses further demonstrated that leptin orchestrates widespread biological reprogramming extending beyond canonical JAK/STAT signaling. In MDA-MB-231 cells, leptin preferentially regulated pathways involved in RNA processing, protein export, steroid biosynthesis, and transcriptional regulation while suppressing AMPK signaling, autophagy, and cell adhesion. Conversely, MDA-MB-468 cells exhibited activation of PI3K-AKT, Notch signaling, extracellular matrix remodeling, migration, and IGF signaling, together with suppression of oxidative phosphorylation and mitochondrial metabolic pathways. Dysregulated RNA splicing can promote tumor plasticity, invasion, and therapeutic resistance [36,37], whereas altered steroid and lipid metabolism may support tumor growth even in hormone receptor-negative disease [38]. The consistent inhibition of AMPK signaling observed in both cell lines is particularly noteworthy, as AMPK serves as a central metabolic checkpoint that restricts tumor growth under nutrient stress [39]. Suppression of this pathway may therefore represent an important mechanism through which leptin facilitates metabolic adaptation in obesity-associated BC. Leptin-mediated activation of ECM-receptor interactions, PI3K/AKT, Notch, cell adhesion, and migration is consistent with mechanisms implicated in TNBC aggressiveness, stemness, and therapeutic resistance [40,41,42]. Collectively, these findings suggest that leptin may promote TNBC progression through distinct combinations of oncogenic and metabolic programs depending on cellular context.

A substantial proportion of leptin-responsive genes identified in cultured TNBC cells displayed concordant expression patterns in TCGA breast tumors and corresponding changes in protein expression in the Human Protein Atlas (HPA), providing complementary evidence for their potential pathobiological relevance. It is, however, worth noting that the HPA data do not establish that these expression patterns result solely from leptin-mediated signaling but rather emphasize their pathobiological significance in breast cancer. Expression of these genes may be influenced by several intrinsic or extrinsic regulatory mechanisms, including leptin signaling. The agreement between experimental and patient-derived datasets substantially increases confidence that these transcriptional changes are biologically relevant rather than culture-specific artifacts. Importantly, many validated genes have established or emerging roles in cancer biology. For example, *LHX2* has been implicated in stemness and tumor progression [43,44,45]; *ABCG1* regulates cholesterol transport and metabolic homeostasis [46,47]; *F2RL3/PAR4* contributes to inflammatory signaling [48]; and *ITGAX* participates in immune regulation and cell migration [49,50]. The functional contributions of several newly identified leptin-responsive genes remain unknown and warrant future mechanistic investigations.

Our analyses also identified race-associated differences in the expression of several leptin-responsive genes in patient tumors. *RORC*, *RTN4RL1*, *ABCG1*, *MAGI2*, and *TRIM68* showed higher expression in Caucasian tumors, whereas *ALPL*, *COL23A1*, *HAAO*, *SLC2A4*, and *POM121L9P* were more highly expressed in tumors from African American patients. These differences suggest that leptin-responsive molecular programs may intersect with tumor biology associated with racial disparities. For example, *ABCG1* is involved in lipid homeostasis [46], whereas *SLC2A4*/*GLUT4* regulates glucose transport and cellular metabolism [51]. As obesity, insulin resistance, and metabolic dysfunction are associated with TNBC risk and disparities [16,52], these genes warrant further investigation as potential links between systemic metabolic status and tumor-specific biology. While TCGA analysis provides supportive evidence that the transcriptional differences observed in cell-line models may be relevant to race-associated differences in disease biology, these findings need validation, as other patient-level clinical, demographic, and environmental factors may also contribute to the observed differences.

Furthermore, several leptin-responsive genes were associated with overall survival, providing additional clinical relevance. Higher expression of leptin-induced *LHX2*, *KCNK1*, *ABCG1*, and *TMEM105* was associated with poorer survival, whereas expression of leptin-suppressed *DIRAS3* and *DCHS2* was associated with more favorable outcomes. These survival associations, together with the concordance observed between cell-based, tumor, and protein-expression datasets, support the potential prognostic relevance of selected leptin-responsive genes. It is, however, to be noted that the observed transcriptional differences between MDA-MB-231 and MDA-MB-468 cells cannot be attributed solely to racial differences, as these cell line models differ in several other intrinsic molecular and biological characteristics. Nevertheless, several of the cell-line-specific genes identified in our analysis also exhibited differential expression between CA and AA patients in the TCGA BC dataset, providing supportive evidence for their potential relevance to race-associated transcriptional differences in BC. Future studies incorporating multiple cell lines and patient-derived models from diverse racial backgrounds will be important for firmly establishing race-specific molecular differences.

Overall, our findings identify leptin as a potential regulator of heterogeneous transcriptional programs in TNBC involving oncogenic signaling, metabolism, inflammation, and tumor-microenvironment interactions. The convergence of leptin-responsive genes with patient tumor expression, race-associated molecular patterns, protein expression, and survival outcomes provides a translational framework linking obesity-associated leptin signaling to TNBC biology. These findings warrant functional validation, particularly in the context of obesity-associated TNBC and breast cancer disparities.

## 4. Methods

### 4.1. Study Cohort, Sample Processing, and Leptin Measurement

The study cohort included women (18 years or older) who self-reported as Black or White and had a confirmed BC diagnosis with no prior history of any other cancer. Black and White women without a BC diagnosis were also recruited for comparative studies. Written informed consent was obtained from all volunteers, the study protocol was approved by the Institutional Review Board (IRB-1143193), and all relevant regulations and guidelines were followed. Blood samples were processed and subjected to leptin measurement as previously described [16].

### 4.2. Cell Culture and Treatment Conditions

Human BC cell lines (MDA-MB-231, MDA-MB-468, MDA-MB-453, and HCC1806) were purchased from the American Type Culture Collection (ATCC) and cultured in Dulbecco’s Modified Eagle Medium (DMEM) and Roswell Park Memorial Institute (RPMI) media; HCC1806 cells (Corning, Glendale, AZ, USA) were cultured at 37 °C in a humidified incubator with 5% CO2. The cell culture medium was supplemented with heat-inactivated Fetal Bovine Serum (FBS; 10%; R&D Systems, Minneapolis, MN, USA) and penicillin/streptomycin antibiotic (1×; Gibco, Grand Island, NY, USA). Short tandem repeat genotyping and intermittent testing for cell line-specific marker proteins were performed to validate the authenticity of the cell lines. To check the expression of p-STAT3 and STAT3, both cell lines were treated with different concentrations of leptin (R&D Systems, MN, USA) (0–200 ng/mL) for 30 min. For RNA sequencing, both cell lines were treated with vehicle or leptin (100 ng/mL) for 24 h at sub-confluence, followed by RNA isolation and sequencing. Cell lines were routinely tested for mycoplasma contamination using the MycoAlert Mycoplasma Detection Kit (Lonza, Walkersville, MD, USA) prior to treatment.

### 4.3. RNA Isolation, Library Preparation, and Next-Generation Sequencing

RNA isolation, library preparation, and next-generation sequencing were performed by the Molecular and Genomics Core Facility at the University of Mississippi Medical Center (Jackson, MS, USA). Total RNA was isolated from cells using the PureLink RNA Mini Kit with TRIzol Reagent (Invitrogen/Life Technologies, Carlsbad, CA, USA) according to the manufacturer’s protocol. RNA quality and integrity were assessed using the Agilent TapeStation 4200 (Agilent Technologies, Santa Clara, CA, USA), and RNA concentration was quantified using a Qubit Fluorometer (Thermo Fisher Scientific, Waltham, MA, USA). All samples met quality thresholds for library preparation, with RNA Integrity Number (RIN) values ranging from 8.6 to 10.0. Strand-specific libraries were constructed from up to 100 ng of total RNA per sample using the TruSeq Stranded mRNA Library Prep Kit (Illumina; San Diego, CA, USA) per the manufacturer’s protocol, which includes poly-A selection for mRNA enrichment. The library was sequenced on the Illumina NextSeq 2000 using P3 (200 cycles, paired-end 100 bp). The run generated ~2.3 billion reads (%QC30 = 93.91) and was assessed for quality using FastQC (version 0.12.1) [53]. The RNA-seq data in this study were deposited in the Gene Expression Omnibus (GEO) database under accession ID: GSE349027.

### 4.4. RNA-Seq Data Analysis

The raw reads were aligned using the STAR aligner (STAR-2.7.10a_alpha_220818) to the human reference genome (GRCh38) with an Ensembl annotation GTF file (Homo_sapiens.GRCh38.113.gtf, version 113) [54]. Alignment was performed with default parameters, and uniquely mapped reads were retained to generate count data using FeatureCounts (2.0.2) [55]. Initial processing and data normalization were performed using the built-in normalization methods in the DESeq2 package in R 4.5.1, which account for differences in library sizes and biological variability [56,57]. In initial processing, genes were retained if they had at least 10 raw read counts in at least three samples across the dataset. This filtering criterion was implemented in R. Genes that did not meet it were excluded prior to downstream differential expression analysis. Genes with zero counts in individual samples were retained if they satisfied the filtering criterion, whereas genes with zero counts across all samples were excluded. Genes with a *p*-value below the specified threshold (*p*-value < 0.05) were considered statistically significant. Volcano plots and DEGs heatmaps were created using SRplot online tools [58].

### 4.5. Quantitative Real-Time PCR (qRT-PCR)

To validate the expression of randomly selected genes after sequencing, Total RNA from cells was isolated using the mirVana RNA isolation kit (Invitrogen, Vilnius, Lithuania) according to the manufacturer’s protocol. RNA was quantified using Nanodrop UltraC (ThermoScientific, MA, USA). Isolated RNA was subjected to reverse transcription using a high-capacity cDNA reverse transcription kit (Applied Biosystems, Foster City, MA, USA). For mRNA expression analysis, 1 μg total RNA was reverse-transcribed using random primers. Quantitative real-time PCR (qRT-PCR) was performed using Maxima SYBR Green qPCR master mix (Thermo Fisher Scientific, MA, USA) on a CFX96 real-time PCR system (Bio-Rad, CA, USA). *ACTB* was used to normalize mRNA expression data. Primer sequences that were used are provided in Table S4.

### 4.6. Western Blot

The cells were processed for Western blot analysis as described earlier [59,60]. Briefly, cell lysates were prepared in RIPA buffer supplemented with protease inhibitor cocktail (100 ×) (Thermo Scientific, Rockford, IL, USA). Cell lysates were sonicated (amplitude 20%, 15 s) followed by centrifugation at 10,000 rpm for 10 min at 4 °C. Protein quantification was done using the DC protein assay kit (Bio-Rad, Hercules, CA, USA). Total protein (40–60 μg) was resolved on SDS-PAGE and transferred onto a PVDF membrane (0.45 μm) (Thermo Scientific, Rockford, IL, USA). The membrane was blocked using 5% skimmed milk in 1× PBST for 1 h. The blocked membrane was incubated with the antibody against the target proteins at a 1:1000 dilution in 5% milk-PBST overnight at 4 °C. Primary antibodies used were OB-R (Invitrogen-PA1-053), p-STAT3 (Cell Signaling Technology, (CST, Danvers, MA, USA)-D3A7), and STAT3 (CST-124H6). Washing was performed using PBST (3X) for 10 min each. HRP-conjugated secondary antibody was added to the membrane at 1:2000 in 5% milk-PBST and incubated for 1 h. Secondary antibodies used were obtained from R&D system (HAF007 and HAF008). After secondary antibody incubation, the membrane was washed (3X) with PBST for 10 min each. Super-Signal West Femto Maximum sensitivity substrate kit (Thermo Scientific, MA, USA) was used to detect protein signal and target protein bands were visualized by ChemiDoc system (Bio-Rad Laboratories, Inc., Hercules, CA, USA). Beta-Actin antibody was procured from CST (12262S).

### 4.7. Pathway Enrichment Analysis

Kyoto Encyclopedia of Genes and Genomes (KEGG) pathway analyses and Gene Ontology (GO) analyses of significantly upregulated and downregulated genes obtained after leptin treatment were performed using the Enrichr web tool [61]. The significant differentially expressed genes were submitted separately as upregulated and downregulated gene lists to identify biological processes and pathways associated with the observed transcriptional changes. KEGG and GO Graphs for significant pathways (*p* < 0.05) were generated using the SRplot online tool [58] and GraphPad Prism (GraphPad Software, version 11.02, San Diego, CA, USA).

### 4.8. Gene and Protein Expression Analysis and Correlation with Tumor Subtype and Patient Survival

Differential expression between normal and BC cases and among various molecular subtypes was analyzed using the TCGA database via the UALCAN platform [22,62]. The Human Protein Atlas (HPA) database was also queried to assess differential protein-level expression [63,64]. To compare leptin-responsive genes and other cellular stress responses, gene sets associated with oxidative stress and hypoxia were downloaded from the ASTRA [35]. Kaplan–Meier survival curves assessing the relationship between gene expression and overall survival were obtained from the TCGA database via the UALCAN platform [62].

### 4.9. Statistical Analysis

RNA-seq experiments were performed in three independent biological replicates. All statistical analyses were conducted using GraphPad Prism, unless otherwise mentioned. The descriptive statistics were computed to calculate the mean (SD) and median (IQR). Fisher’s exact test was used to calculate odds ratios (ORs) and 95% CIs for Black vs. White women across BC subtypes. Serum leptin levels were measured in triplicate, and the 2-tailed unpaired *t*-test was performed to compare levels between the BC and non-BC (control) groups. RNA quantitation experiments were performed at least three times, and data were expressed as mean ± SD. Comparisons between control and treated groups were performed using one-way ANOVA. A *p*-value of <0.05 was considered statistically significant.

## 5. Conclusions

In conclusion, this study showed the important role of leptin signaling in shaping the molecular landscape of TNBC by regulating pathways involved in tumor progression, metabolic adaptation, inflammation, and survival. The distinct transcriptional responses observed between TNBC cell lines derived from Black and White patients highlight the biological heterogeneity of obesity-associated breast cancer and suggest that leptin-mediated signaling may contribute to racial disparities in disease aggressiveness and outcomes. Integration of transcriptomic, clinical, protein expression, and survival analyses identified several clinically relevant leptin-responsive genes as potential therapeutic targets in TNBC. Furthermore, the strong association between leptin-induced DEGs and poor patient survival revealed the translational significance of obesity-driven molecular alterations in aggressive breast cancer. Overall, these findings provide important mechanistic insights into the role of leptin in TNBC progression and support the development of leptin-targeted therapeutic strategies to improve outcomes and reduce disparities in obesity-associated TNBC. Further studies using in vivo models and larger, independently validated patient cohorts are needed to confirm the biological and clinical significance of these leptin-associated molecular alterations and to establish their causal role in TNBC progression and racial disparities.

## Data Availability

All transcriptomic datasets supporting the findings of this study have been deposited in the GEO database under accession ID: GSE349027. Data and materials used during the current study are available from the corresponding authors upon reasonable request.

## Supplementary Materials

The following supporting information can be downloaded at: https://www.mdpi.com/article/10.3390/ijms27219680/s1.
