# Supplementary material for: Leptin-Driven Distinct Transcriptional Reprogramming in Triple-Negative Breast Cancer: Implications for Racial Disparities
Source: Int J Mol Sci. 2026 Oct 29;27(21):9680. doi: 10.3390/ijms27219680 (PMC13762763; doi:10.3390/ijms27219680)
Supplement: Supplementary file 1 [file ijms-27-09680-s001.zip › ijms-4629385-supplementary.pdf]

Leptin-Driven Distinct Transcriptional Reprogramming in Triple-Negative Breast Cancer: Implications for Racial Disparities

Sarabjeet Kour Sudan[1,2,*], Amod Sharma[1,2], Shashi Anand[1,2], Lavanya Challagundla[1], Jasleen Kaur[1], Barbara S. Craft[2,3], Ajay P. Singh[1,2], and Seema Singh[1, 2, *]

[1]Department of Cell and Molecular Biology, University of Mississippi Medical Center, Jackson, Mississippi, USA

[2]Cancer Center and Research Institute, University of Mississippi Medical Center, Jackson, Mississippi, USA

[3]Division of Oncology, University of Mississippi Medical Center, Jackson, Mississippi, USA

*Correspondence: ssingh3@umc.edu and ssudan@umc.edu

***Corresponding Authors**

Seema Singh, PhD, MBA
Professor, Cell and Molecular Biology
Associate Director of Education and Training
Cancer Center and Research Institute
University of Mississippi Medical Center
2500 North State Street, Jackson, MS 39216
Email: ssingh3@umc.edu

Sarabjeet Kour Sudan, PhD
Instructor, Cell and Molecular Biology
Associate Member,
Cancer Center and Research Institute
University of Mississippi Medical Center
2500 North State Street, Jackson, MS 39216
Email: ssudan@umc.edu

Supplementary Table S1. RNA-seq quality control metrics for all samples.

Sample	Total reads	Avg. input read length	Unique reads	Unique Mapping (%)	Multi Mapped reads	Mismatch Rate (%)	Avg. mapped length
MDA-MB-231 Control 1	39533218	199	34208100	86.53%	2407695	0.23%	199.27
MDA-MB-231 Control 2	41694514	196	36284586	87.02%	2811359	0.23%	196.04
MDA-MB-231 Control 3	41010647	196	35381624	86.27%	2809681	0.23%	196.05
MDA-MB-231 Treatment 1	36485739	199	32007991	87.73%	2267716	0.23%	198.98
MDA-MB-231 Treatment 2	38008154	199	33534155	88.23%	2406253	0.23%	198.88
MDA-MB-231 Treatment 3	35636336	197	31116238	87.32%	2382384	0.23%	196.84
MDA-MB-468 Control 1	41694181	198	36154093	86.71%	2762740	0.24%	198.4
MDA-MB-468 Control 2	33808428	199	29729248	87.93%	2122274	0.24%	199.38
MDA-MB-468 Control 3	40770629	197	36060810	88.45%	2857338	0.24%	197.14
MDA-MB-468 Treatment 1	45694246	198	39884147	87.28%	3132013	0.24%	197.97
MDA-MB-468 Treatment 2	32791609	200	28518984	86.97%	1947471	0.24%	199.54
MDA-MB-468 Treatment 3	33226140	199	28655864	86.24%	2015639	0.25%	199.45

Supplementary Table S2. Up regulated and downregulated KEGG pathways associated genes in MDA-MB-231 and MDA-MB-468 cells.

**Pathways**	**Genes**
**MDA-MB-231 upregulated DEGs**	
Spliceosome	*SF3A3*;*DDX5*;*PRPF38B*;*HNRNPA3*;*DDX46*;*SF3B6*;*CWC15*;*PLRG1*;*LSM5*;*USP39*;*LSM8*;*SNRNP40*;*SNW1*;*PRPF18*;*SYF2*;*SLU7*;*SRSF10*;*RBMX*;*BCAS2*
Steroid biosynthesis	*SQLE*;*SC5D*;*MSMO1*;*LIPA*;*LBR*
Protein export	*SRP19*;*HSPA5*;*SRP72*;*SRP54*;*SEC11A*
Basal transcription factors	*GTF2A2*;*GTF2H2C*;*TAF12*;*GTF2B*;*TAF11*;*TAF9B*;*GTF2F2*
Pyruvate metabolism	*GLO1*;*ME1*;*PDHB*;*ALDH7A1*;*DLD*;*ACAT2*;*ACAT1*
**MDA-MB-231 downregulated DEGs**	
AMPK signaling pathway	*SREBF1*;*PFKFB3*;*IRS1*;*PPP2R5B*;*PIK3CD*;*IRS2*;*SLC2A4*;*EEF2*;*RPTOR*;*GYS1*;*PFKL*;*FASN*;*ULK1*
Focal adhesion	*LAMA5*;*JUN*;*LAMB3*;*ITGB4*;*ITGA3*;*ACTN1*;*PXN*;*PIK3CD*;VAV1;COL6A2;*RAPGEF1*;*FLNA*;*PAK6*;*PIP5K1C*;*FLNC*;*TLN1*
Longevity regulating pathway	*RPTOR*;*CLPB*;*SESN3*;*ATF6B*;*IRS1*;*EHMT2*;*PIK3CD*;*IRS2*;*ULK1*;*ADCY6*
Insulin signaling pathway	*RPTOR*;*GYS1*;*SREBF1*;*PRKAR1B*;*IRS1*;*FASN*;*INPPL1*;*RAPGEF1*;*PIK3CD*;*IRS2*;*SLC2A4*;*PTPRF*
Endocytosis	*GBF1*;*VPS37D*;*AP2A1*;*VPS37B*;*EPN1*;*PML*;*EHD1*;*EHD2*;*GRK2*;*HGS*;*CHMP1A*;*MVB12A*;*PIP5K1C*;*CHMP6*;*SMAP2*;*FGFR3*;*GIT1*;*SH3GL1*
**MDA-MB-468 upregulated DEGs**	
ECM-receptor interaction	*ITGB4*;*ITGA3*;*ITGB3*;*FN1*;*COL6A3*;*LAMC1*;*ITGA5*;*HSPG2*;*THBS1*
Notch signaling pathway	*NOTCH2*;*NOTCH3*;*CREBBP*;*ATXN1*;*EP300*;*DTX2*;*DTX4*
PI3K-Akt signaling pathway	*PHLPP2*;*MAGI1*;*PHLPP1*;*IL4R*;*IRS1*;*ITGB4*;*ITGA3*;*INSR*;*ITGB3*;*FN1*;*LAMC1*;*FOXO3*;*THBS1*;*EGFR*;*PIK3CG*;*IGF1R*;*COL6A3*;*ITGA5*
Transcriptional misregulation in cancer	*BCL6*;*PAX8*;*H3C13*;*TFE3*;*H3C12*;*NSD2*;*RARA*;*HMGA2*;*IGF1R*;*RUNX1*;*KDM6A*
Pathways in cancer	*NOTCH2*;*STAT5A*;*PTGER4*;*NOTCH3*;*CREBBP*;*IL4R*;*DAPK1*;*ITGA3*;*HHIP*;*PTCH1*;*FN1*;*LAMC1*;*KIF7*;*EGFR*;*RUNX1*;*IGF1R*;*SHH*;*PAX8*;*CDH1*;*SUFU*;*RARA*;*EP300*;*IL12A*;*F2RL3*

**MDA-MB-468 downregulated DEGs**	
Oxidative phosphorylation	*ATP5PF*;*NDUFB8*;*ATP5PD*;*UQCRB*;*NDUFA12*;*NDUFB5*;*ATP5PB*;*NDUFB2*;*NDUFA1*;*UQCR11*;*COX6A1*;*COX5B*;*COX6C*;*PPA2*;*UQCRQ*;*NDUFS5*;*NDUFS4*;*NDUFAB1*;*ATP5MG*
mRNA surveillance pathway	*PPP2CA*;*NXT1*;*CPSF3*;*CSTF2*;*PPP2R2D*;*PPP2R2A*;*PPP2R5C*;*MAGOHB*
Citrate cycle (TCA cycle)	*FH;SUCLA2*;*SUCLG1*;*PDHB*
AMPK signaling pathway	*PPP2CA*;*CREB3L1*;*PPP2R2D*;*PRKAG2*;*PPP2R2A*;*PPARG*;*PPP2R5C*;*PPARGC1A*
Folate biosynthesis	*MOCS2*;*PCBD1*;*PTS*

Supplementary Table S3. Up regulated and downregulated GO biological pathways associated genes in MDA-MB-231 and MDA-MB-468 cells.

Pathways	Genes
**MDA-MB-231 upregulated DEGs**	
RNA Splicing, via Transesterification Reactions With Bulged Adenosine as Nucleophile (GO:0000377)	*ZCCHC8*;*DDX5*;*DDX46*;*YTHDC1*;*SF3B6*;*CWF19L2*;*HTATSF1*;*USP39*;*PNN*;*CWC22*;*SNIP1*;*MTREX*;*SRSF10*;*BCAS2*;*SF3A3*;*HNRNPA3*;*CENATAC*;*CWC15*;*PLRG1*;*LSM5*;*DHX40*;*LSM8*;*SNRNP40*;*SNW1*;*SYF2*;*PPIL3*;*SLU7*;*RBMX*
mRNA Processing (GO:0006397)	*ZCCHC8*;*DDX5*;*DDX46*;*YTHDC1*;*SF3B6*;*CWF19L2*;*HTATSF1*;*USP39*;*CDC73*;*PNN*;*CWC22*;*SNIP1*;*MTREX*;*SRSF10*;*BCAS2*;*SF3A3*;*HNRNPA3*;*WTAP*;*CENATAC*;*CWC15*;*PLRG1*;*LSM5*;DHX40;*KIN*;*LSM8*;*SNRNP40*;*SNW1*;*PRPF18*;*SYF2*;*PPIL3*;*SLU7*;*RBMX*
Protein Transport (GO:0015031)	*RAB1A*;*ZNF395*;*TMED10*;*SAR1A*;*VPS26A*;*VLDLR*;*HSP90B1*;*RPGR*;*SCFD1*;*TIMM17A*;*TMED2*;*STX3*;*RAB6A*;*SNX6*;*BBS2*;*NPM1*;*VPS33A*;*STX7*;*CMTM6*;*TVP23B*;*RAB11A*;*VMP1*;*RAB10*;*ARL4A*;*SNX15*;*CHMP2B*;*PPT1*;*SLU7*;*VAMP5*;*RAB5A*;*BLZF1*;*ABCG1*
Protein Ubiquitination (GO:0016567)	*RNF13*;*RNF14*;*CUL3*;*FBXO25*;*RCHY1*;*CCAR1*;*UBE2J1*;*RPGR*;*HERC5*;*CAND1*;*UBE2Q1*;*TRIM2*;*FBXO4*;*FBXO9*;*RNF20*;*ATG3*;*CRBN*;*FBXW11*;*UBE2E1*;*KLHL2*;*WWP1*;*TOPORS*;*UBE2W*;*RNF225*;*KLHL7*;*RFWD3*;*UBE2T*;*RNF228*;*MKRN1*;*SDE2*;*UBE2V2*;*UBE2N*;*ERCC8*;*NHLRC1*;*TRIM37*;*NFE2L2*
Sterol Biosynthetic Process (GO:0016126)	*SQLE*;*HMGCS1*;*PRKAG2*;*MSMO1*;*HMGCR*;*LBR*
**MDA-MB-231 downregulated DEGs**	
Positive Regulation of Nucleocytoplasmic Transport (GO:0046824)	*TGFB1*;*TRIM28*;*JUP*;*BAG3*;*ZC3H12A*;*EP300*;*FLNA*;*HCLS1*;*SIRT6*

Regulation of Actin Cytoskeleton Organization (GO:0032956)	*CSF1R*;*CSF3*;*INPPL1*;*TWF2*;*HIP1R*;*SLC4A2*;*RHOF*;*CELSR1*;*BAIAP2*;*PEAK3*;*SH3BP1*;*TAOK2*;*FSCN1*;*MLST8*
Positive Regulation of Autophagy (GO:0010508)	*PINK1*;*SESN3*;*ZC3H12A*;*NPRL3*;*ATG2A*;*TFEB*;*ULK1*;*TICAM1*;*SUPT5H*;*WDR24*;*TPCN1*
Receptor-Mediated Endocytosis (GO:0006898)	*HIP1R*;*AP2A1*;*LRP3*;*HSPG2*;*LRP1B*;*TPCN2*;*CXCL16*;*GAK*;*GRK2*;*HGS*;*PLXNB2*;*PIP5K1C*;*PDLIM7*
Cell-Cell Adhesion *via* Plasma-Membrane Adhesion Molecules (GO:0098742)	*ROBO4*;*PTPRS*;*L1CAM*;*CELSR2*;*PKD1*;*FGFRL1*;P*TPRF*;*ICAM1*;*CDH4*;*CLDN4*;*PTK7*;*PLXNB3*;*PLXNB2*;*MPZL2*;*NECTIN1*
**MDA-MB-468 upregulated DEGs**	
Regulation of miRNA Transcription (GO:1902893)	*NOTCH2*;*NOTCH3*;*ATOH8*;*RARA*;*SOX9*;*FOXO*3;*EGFR*;*APLN*;*SMARCA4*
Regulation of Cell-Cell Adhesion Mediated by Integrin (GO:0033632)	*PODXL*;*WNK1*;P*IEZO1*
Insulin-Like Growth Factor Receptor Signaling Pathway (GO:0048009)	*IRS1*;I*RS2*;I*GF1R*
Positive Regulation of MAP Kinase Activity (GO:0043406)	*ERN1*;*INSR*;*DUSP19*;*THBS1*;*PIK3CG*;*ARHGEF5*;*ROBO1*
Positive Regulation of Cell Migration (GO:0030335)	*NRP1*;*NRP2*;*CEMIP*;*INSR*;*ITGB3*;*CLSTN1*;*FN1*;*THBS1*;*EGFR*;*PIK3CG*;*IGF1R*;*FOXP1*;*PODXL*;*MGAT5*;*ZNF703*;*ATOH8*;*ITGAX*;*HAS2*;*SOX9*;*BCAR1*
**MDA-MB-468 downregulated DEGs**	
Cytoplasmic Translation (GO:0002181)	*RWDD1*;*RPL5*;*RPL41*;*RPL21*;*RPS8*;*RPL12*;*RPL35A*;*RPS3A*;*EIF2S2*;*RPL9*;*MRPL13*;*RPS4X*;*RPS25*;*SARS1*;*RPS15A*;*RPS3*;*RPL38*;*RPL27*;*RPS20*;*RPS24*
Oxidative Phosphorylation (GO:0006119)	*ATP5PF*;*NDUFB8*;*ATP5PD*;*UQCRB*;*NDUFA12*;*NDUFB5*;*ATP5PB*;*NDUFB2*;*NDUFA1*;*NDUFS5*;*NDUFS4*;*NDUFAB1*;*ATP5MG*
Ribosome Biogenesis (GO:0042254)	*LTV1*;*RPL5*;*EIF5B*;*NPM1*;*RPS8*;*NGDN*;*NOL7*;*RPL35A*;*RPS3A*;*MALSU1*;*UTP18*;*RPS4X*;*RPS25*;*EMG1*;*RPS15A*;*XRN2*;*HEATR3*;*RPL27*;*PIN4*;*RPS24*;*DCAF13*
mRNA Processing (GO:0006397)	*SF3A3*;*SF3B6*;*CPSF3*;*CSTF2*;*C9ORF78*;*MAGOHB*;*LSM3*;*PRPF4*;*LSM8*;*ZMAT2*;*CWC22*;*SNRPG*;*SNIP1*;*SNRNP27*;*SNRPB2*;*TXNL4A*;SNRPC;*PPARGC1A*;*SRSF9*
protein-RNA Complex Assembly (GO:0022618)	*RPL5*;*XRCC5*;*EIF2S2*;*SHQ1*;*EIF3L*;*SNRPG*;*EIF3H*;*RPL38*;*EIF3E*;*TXNL4A*;*SNRPC*;*EIF3D*;*SRSF9*

Supplementary Table S4. List of primers and their sequence used for quantitative real-time PCR.

S. No.	Primer name	Sequence (5' to 3')
1.	CD207 F	TAATCTGCCTGACGCTGGTCCT
2.	CD207 R	GGTGCTGATGTTGTCCACACGA
3.	RHOH F	TGGATGGCATCCAGATCAGCCT
4.	RHOH R	GCCACAGAGTAGCACATCAGCA
5.	KCNK15 F	GGCTCCTTCTACTTCGCCATCA
6.	KCNK15 R	CTGGAAAGTGACCAGCGTCAGC
7.	SARNP F	TCTTGCTCGTGGTTTGGAGACC
8.	SARNP R	TCTTCCTCTGTTTCATCTCCCAG
9.	RORC F	GAGGAAGTGACTGGCTACCAGA
10.	RORC R	GCACAATCTGGTCATTCTGGCAG
11.	F2RL3 F	CCAGCGTCTACGACGAGAGCG
12.	F2RL3F R	AGCTCCAGGGTGTCACTGTCAT
13.	B4GALNT1 F	TGAGGCTGCTTTCACTATCCGC
14.	B4GALNT1 R	GAGGAAGGTCTTGGTGGCAATC
15.	ITGAX F	GATGCTCAGAGATACTTCACGGC
16.	ITGAX R	CCACACCATCACTTCTGCGTTC
17.	OPRL1 F	AGCAAAGCCCAGGCTGTCAATG
18.	OPRL1 R	ATCTCCACCAGGCACTCGATCT
19.	SYT14 F	CCAAGTGAAGGAAGCACAGGTC
20.	SYT14 R	GCTGCGAAGATGATCCTTGAGC
21.	SLC2A4 F	CCATCCTGATGACTGTGGCTCT
22.	SLC2A4 R	GCCACGATGAACCAAGGAATGG
23.	DLX1 F	CATCAGTTCGGTGCAGTCCTAC
24.	DLX1 R	CCTTGCCATTGAAGCGCACTTC
25.	SRGAP3 F	TTCCGAGGACTGGAAAACCCAC
26.	SRGAP3 R	GGGAAGGGTGACGAGGATTTGT
27.	LPO F	CTGCCCTATGACAGCAAGAAGC
28.	LPO R	CGGTTATGCTCGCGGAGAAAGA

29.	DIRAS3 F	CACATCACCGACAGCAAGAGTG
30.	DIRAS3 R	CAGGGTTTCCTTCTTGGTGACTG
31.	PPARGC1A F	CCAAAGGATGCGCTCTCGTTCA
32.	PPARGC1A R	CGGTGTCTGTAGTGGCTTGACT
33.	SYN2 F	ACCTGCTCTGAGATGTTTGGCG
34.	SYN2 R	GTTCGGTGATGAGTTGCCTGTC
35.	RASD1 F	CACCGCAAGTTCTACTCCATCC
36.	RASD1 R	GGTTGTCCAGACTGAACACCAG
37.	TAGLN3 F	CCTGATGGCTTTAGGCAGCGTT
38.	TAGLN3 R	GGAAAAGCCTCTCCGATTCTGC
39.	SPN F	GCAACCAGTCATCCTGCTGTTC
40.	SPN R	CTGGTCCTACTGGAGGTTTCTG

Supplementary Figure S1

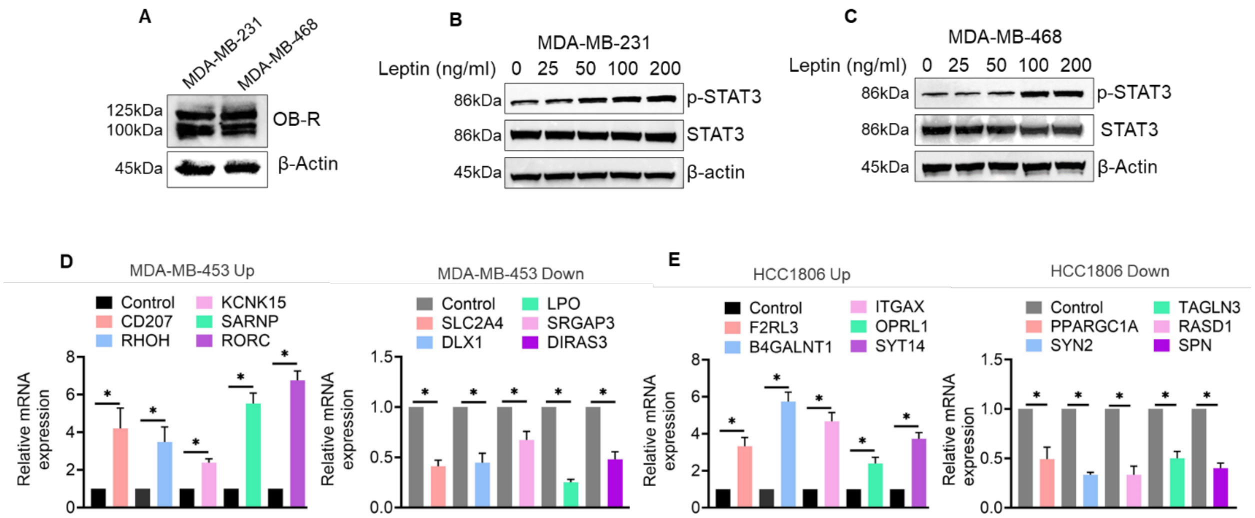

Figure S1. Leptin receptor, pSTAT3 expression, and differential gene expression validation in breast cancer cell lines. (A) Representative immunoblots showing the basal expression of leptin receptor in MDA-MB-231 and MDA-MB-468 cells. (B, C) Dose dependent effect of leptin (0-200ng/ml) on p-STAT3 expression was examined by western blotting in both MDA-MB-231 and MDA-MB-468 cells. β-actin was used as an internal control (D, E) Validation of upregulated and downregulated genes in MDA-MB-453 and HCC1806 cells. Bar graphs showing the relative mRNA expression of genes compared to vehicle treated control. Data are presented as mean ± SD (*p < 0.05).

Supplementary Figure S2

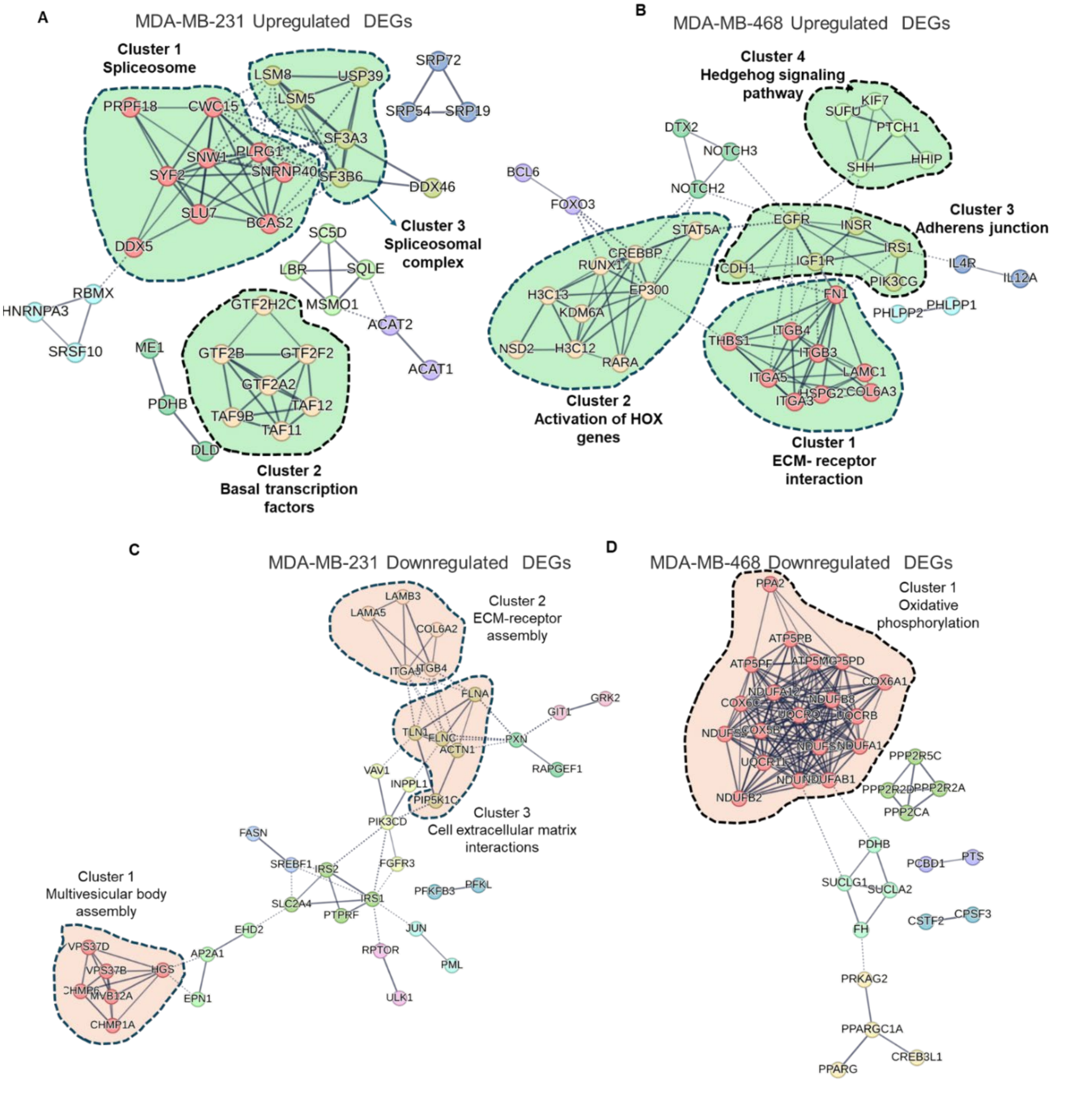

Figure S2. Protein–protein interaction (PPI) network analysis of differentially expressed genes in leptin-treated breast cancer cells. (A, B) PPI network constructed from significantly upregulated differentially expressed genes in leptin-treated MDA-MB-231 and MDA-MB-468 cells. Clustering show functionally related modules. (C, D) PPI network of Downregulated DEGs in leptin-treated MDA-MB-231 and MDA-MB-468 cells.

Supplementary Figure S3

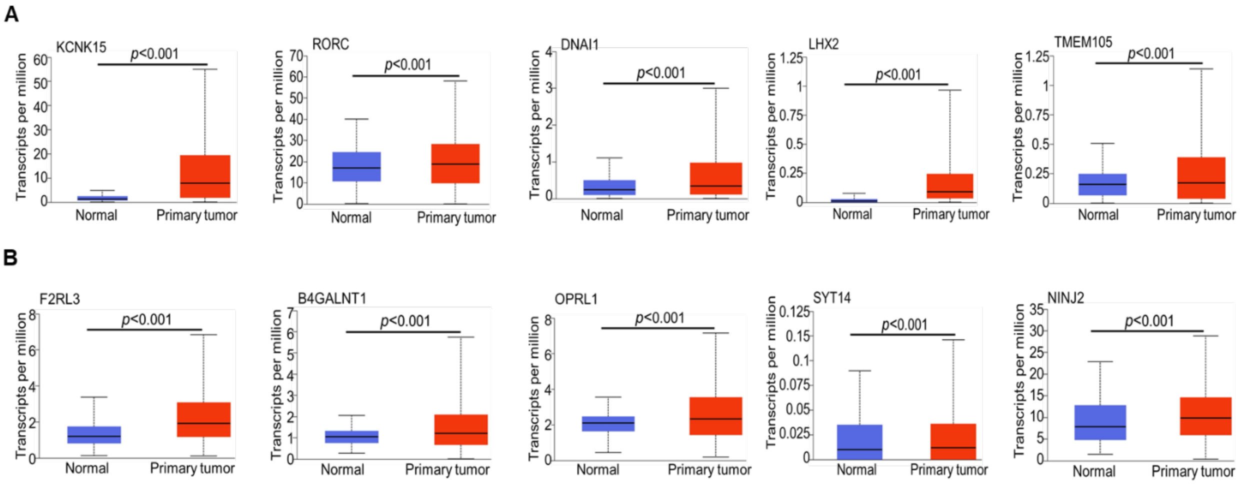

Figure S3. Validation of upregulated genes using TCGA-BRCA patient dataset. (A, B) Expression levels of differentially expressed upregulated genes in MDA-MB-231(A) and MDA-MB-468 (B) cells was independently evaluated in breast tumor and normal tissues using the TCGA-BRCA dataset. Plots show the expression levels of the corresponding genes in tumor tissues (N = 1,097) compared with normal breast tissues (N = 114). Statistical significance for tumor-versus-normal comparisons is indicated above each plot, along with exact p-values.

Supplementary Figure S4

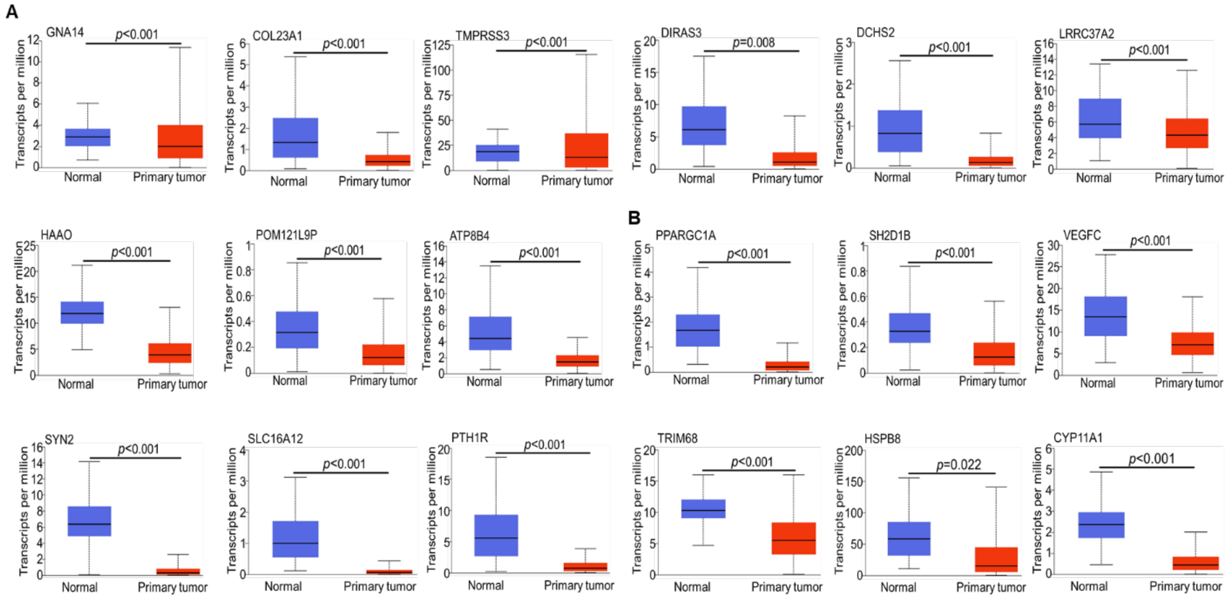

Figure S4. Validation of downregulated genes using TCGA-BRCA patient dataset in UALCAN. (A, B) Expression levels of differentially expressed downregulated genes in MDA-MB-231 (A) and MDA-MB-468 cells (B) was independently evaluated in breast tumor and normal tissues using the TCGA-BRCA dataset. Plots show the expression levels of the corresponding genes in tumor tissues (N = 1,097) compared with normal breast tissues (N = 114). Statistical significance for tumor-versus-normal comparisons is indicated above each plot, along with exact p-values.

Supplementary Figure S5

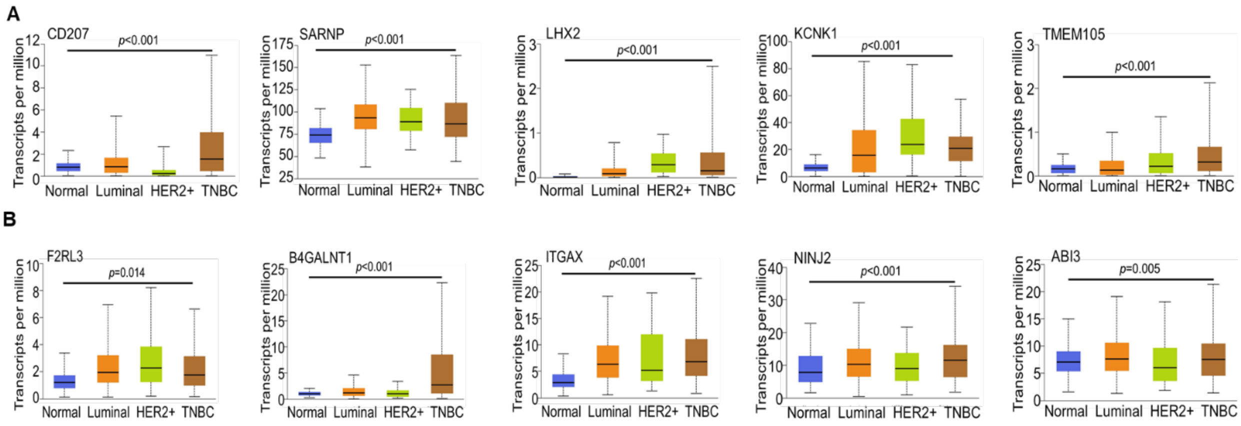

Figure S5. Expression analysis of upregulated genes in normal breast tissue versus the TNBC subtype. (A, B) Expression levels of differentially expressed upregulated genes in MDA-MB-231 (A) and MDA-MB-468 (B) cells was evaluated in breast tumor and normal tissues using the TCGA-BRCA dataset. Plots show the expression levels of the corresponding genes in normal breast tissues (N = 114) compared with TNBC subtype (N = 116) tissue samples. Statistical significance for tumor versus normal comparisons is indicated above each plot, with exact *p*-values.

Supplementary Figure S6

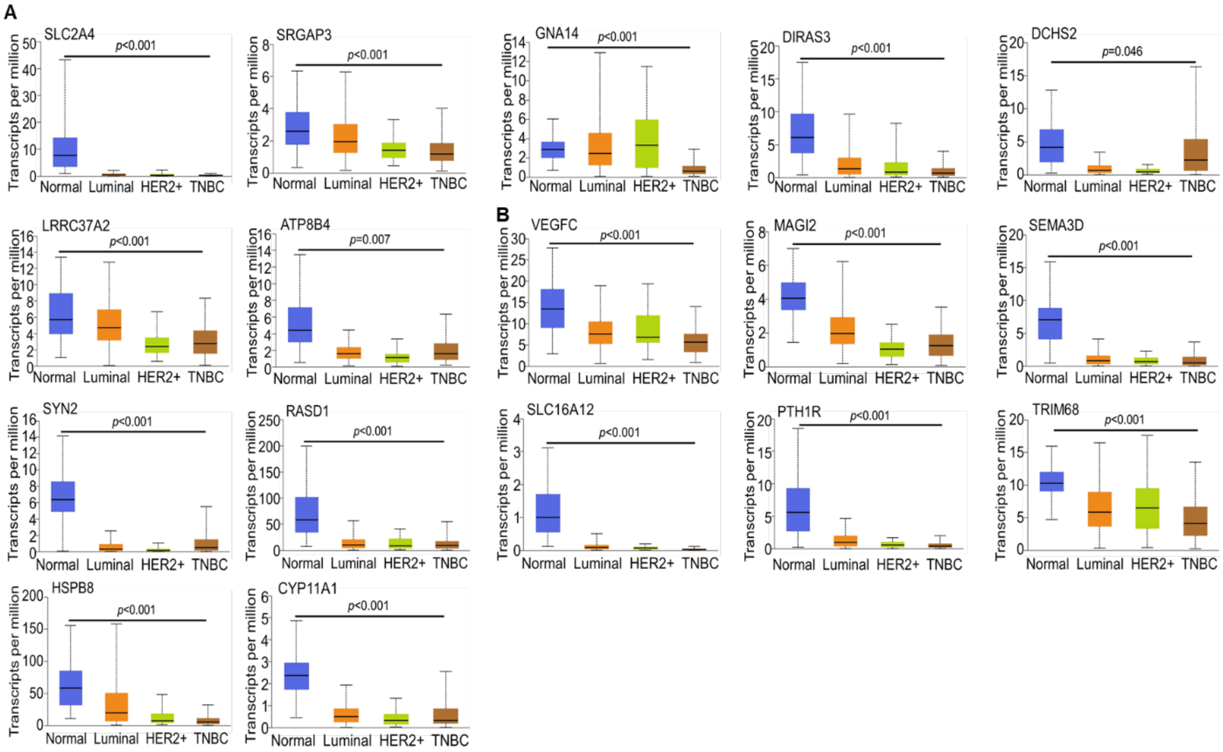

Figure S6. Expression analysis of downregulated genes in normal breast tissue versus the TNBC subtype. (A, B) Expression levels of differentially expressed downregulated genes in MDA-MB-231 (A) and MDA-MB-468 (B) cells was evaluated in breast tumor and normal tissues using the TCGA-BRCA dataset. Plots show the expression levels of the corresponding genes in normal breast tissues ($N = 114$) compared with TNBC subtype ($N = 116$) tissue samples. Statistical significance for tumor versus normal comparisons is indicated above each plot, with exact *p*-values.
